# Enhanced NOLC1 promotes cell senescence and represses hepatocellular carcinoma cell proliferation by disturbing the organization of nucleolus

**DOI:** 10.1111/acel.12602

**Published:** 2017-05-10

**Authors:** Fuwen Yuan, Yu Zhang, Liwei Ma, Qian Cheng, Guodong Li, Tanjun Tong

**Affiliations:** ^1^ Peking University Research Center on Aging Department of Biochemistry and Molecular Biology Peking University Health Science Center, Beijing, Key Laboratory of Protein Posttranslational Modifications and Cell Function Beijing 100191 China; ^2^ Department of Hepatobilliary Surgery Beijing, Key Surgical Basic Research Laboratory of Liver Cirrhosis and Liver Cancer Peking University People's Hospital Beijing 100044 China

**Keywords:** aging, CSIG, hepatocellular carcinoma, NOLC1, nucleolus

## Abstract

The nucleolus is a key organelle that is responsible for the synthesis of rRNA and assembly of ribosomal subunits, which is also the center of metabolic control because of the critical role of ribosomes in protein synthesis. Perturbations of rRNA biogenesis are closely related to cell senescence and tumor progression; however, the underlying molecular mechanisms are not well understood. Here, we report that cellular senescence‐inhibited gene (CSIG) knockdown up‐regulated NOLC1 by stabilizing the 5′UTR of NOLC1 mRNA, and elevated NOLC1 induced the retention of NOG1 in the nucleolus, which is responsible for rRNA processing. Besides, the expression of NOLC1 was negatively correlated with CSIG in the aged mouse tissue and replicative senescent 2BS cells, and the down‐regulation of NOLC1 could rescue CSIG knockdown‐induced 2BS senescence. Additionally, NOLC1 expression was decreased in human hepatocellular carcinoma (HCC) tissue, and the ectopic expression of NOLC1 repressed the proliferation of HCC cells and tumor growth in a HCC xenograft model.

## Introduction

The nucleolus is a key organelle that coordinates the synthesis of rRNA and the assembly of ribosomal subunits (Andersen *et al*., 2005), which play an important role in protein metabolism (Boisvert *et al*., 2007). NOLC1 was first identified as a nuclear localization signal‐binding protein that also functions as a chaperone for shuttling between the nucleolus and cytoplasm (Meier & Blobel, 1990; Meier & Blobel, 1992). The orthologous of NOLC1 in the human, Xenopus, Drosophila, and worm genomes shares a similar organization and contains conserved *N*‐terminal and *C*‐terminal domains and a central region consisting of several interspersing repeats of acidic and basic amino acid clusters (Cairns & McStay, 1995; Pai *et al*., 1995; Waggener & DiMario, 2002; Lee, 2004). Ubiquitylation drives the formation of a TCOF1‐NOLC1 platform could remodel the translational program of differentiating cells in favor of neural crest specification (Werner *et al*., 2015), and it also acts as a transcriptional regulator to activate the alpha‐1‐acid glycoprotein (AGP) in mammalian livers (Miau *et al*., 1997). In addition, hNOLC1 has been demonstrated to function as a binding target of doxorubicin, a widely used anticancer drug (Jin *et al*., 2002). NOLC1 can also bind to and inhibit the catalytic subunit of CK2 *in vitro* (Li *et al*., 1997; Lee *et al*., 2013). NOLC1 localizes to nucleolar DFCs and participates in the regulation of rRNA transcription by interacting with the largest subunit of RNA Pol I (RPA194) (Chen *et al*., 1999; Tsai *et al*., 2008). Interestingly, over a decade ago, a report indicated that the ring structures were induced in nucleoli by the exogenous expression of NOLC1 (Isaac *et al*., 1998, 2001); however, the underlying mechanisms and biological role remain largely unknown.

Cellular senescence‐inhibited gene (CSIG) was cloned from human diploid fibroblast 2BS cells in our laboratory (GenBank accession no. AY154473) (Guo *et al*., 2004), and it is abundantly expressed in growing human diploid fibroblast cells, although its expression declines during cellular senescence (Guo *et al*., 2004; Ma *et al*., 2008). In addition, CSIG regulates the activity of nucleostemin, which delays the progression of aging in mouse fibroblasts (Meng *et al*., 2006; Zhu *et al*., 2006), and it is required for p33ING1 to induce apoptosis under UV irradiation (Li *et al*., 2012). Furthermore, CSIG is highly expressed in HCC and promotes the HCC cell proliferation (Cheng *et al*., 2015). Although CSIG is implicated in various processes, the regulatory mechanism underlying its functions is not well understood.

In our microarray analysis, we found that NOLC1 was significantly up‐regulated following CSIG knockdown in 293 cells. The aim of this study was to investigate the mechanism by which CSIG impacts the expression of NOLC1 and determine the biological roles of the elevated expression of NOLC1.

## Results

### CSIG affects NOLC1 expression

**Figure 1:**
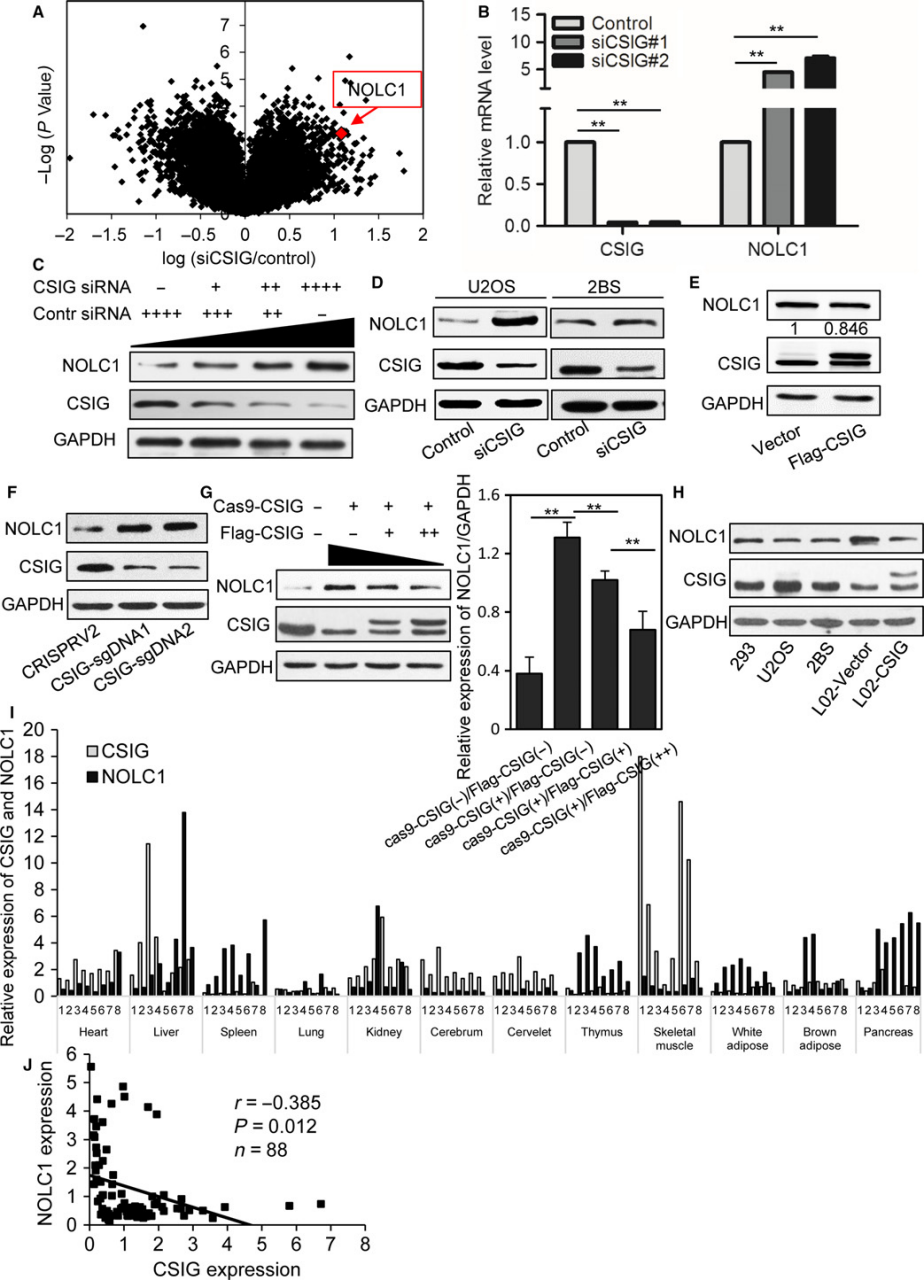
CSIG knockdown up‐regulates NOLC1, and CSIG expression is negatively correlated with NOLC1 *in vivo*. (A) Volcano plot showing the gene expression profiles in the control and CSIG knockdown microarray data. The location of NOLC1 is marked in red. (B) qPCR analysis of the mRNA expression of NOLC1 with CSIG knockdown. (C) Western blot showing the expression of NOLC1 transfected with different amount of CSIG siRNA (0 μm, 5 μm, 25 μm, 50 μm) in 293 cells. (D and E) NOLC1 expression assessed by Western blotting after CSIG knockdown or overexpression. (F) CRISPRV2‐sgDNAs targeting the CSIG up‐regulated NOLC1. (G) CSIG overexpression in CSIG ablated cells repressed the expression of NOLC1 in a dose‐dependent manner. Histogram represented the quantification of the relative expression of NOLC1. (H) Western bolt indicated that the expression of NOLC1 in L02 was more abundant than other cell lines, and the enhanced CSIG inhibited the expression NOLC1 in L02 cells. (I) Relative expression of CSIG and NOLC1 in mouse tissues. Eight mice were used for rRNA extraction and expression assays. (J) Association analysis between CSIG and NOLC1 expression in mouse tissues (*r* = 0.385, *P* = 0.012, *n* = 88). Error bars indicate the SD; **P < 0.01.

To screen important targets and signaling pathways modulated by CSIG, we compared the gene expression profiles in CSIG‐silenced and control 293 cells and validated the profiles via quantitative reverse transcription polymerase chain reaction (qRT–PCR) (Fig. S1). Volcano plot analyses revealed changes in the expression of many genes, and the NOCL1 gene aroused our attention because of its interesting role in the synthesis of rRNA (Fig. 1A). qRT–PCR and Western blot analyses demonstrated that CSIG knockdown increased the expression of NOLC1 (Fig. 1B and C), as well as in human osteosarcoma U2OS and diploid fibroblast 2BS cells (Fig. 1D). However, overexpression of CSIG in 293 cells did not have an apparent influence on NOLC1 expression (Fig. 1E). The abundant background expression of CSIG in 293 cells suggests that CSIG may maintain the expression of NOLC1 at a relatively low level. We then ablated the expression of CSIG in 293 cells using the CRISPRV2–cas9 system to target CSIG (Fig. 1F). The data showed that following the transfection of different amounts of exogenous CSIG in these CSIG ablated cells, the increased NOLC1 induced by CSIG was inhibited to varying degrees (Fig. 1G). Furthermore, in L02 cells which has a low level of CSIG (Cheng *et al*., 2015), the overexpression of CSIG could clearly repress the expression of NOLC1 (Fig. 1H).

### CSIG expression is negatively correlated with NOLC1 *in vivo*

To further explore the relationship between CSIG and NOLC1, we examined the expression of CSIG and NOLC1 in different mouse tissues, including the heart, liver, spleen, lung, kidney, cerebrum, cerebellum, thymus, muscle, adipose, and pancreas of eight BALB/c mice. Our results showed that the expression of CSIG was negatively correlated with NOLC1 in most organs except the kidney (Fig. 1I and J), indicating that CSIG may regulate the expression of NOLC1 *in vivo*.

### CSIG represses NOLC1 by destabilizing NOLC1 mRNA

**Figure 2:**
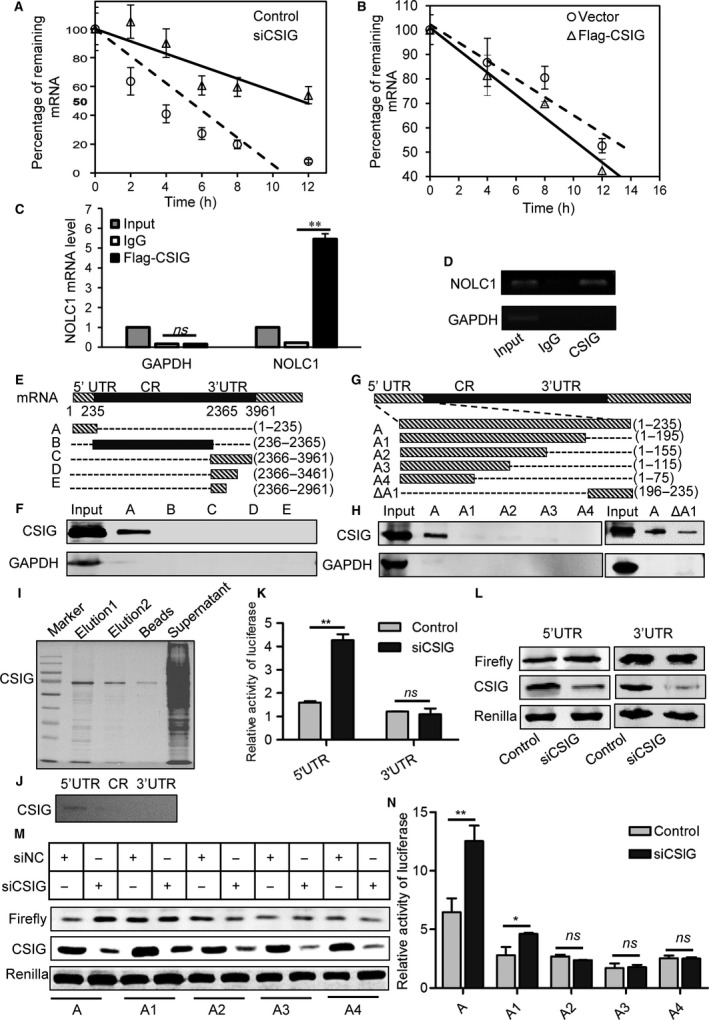
CSIG promotes NOLC1 mRNA degradation depending on the interaction with NOLC1 5′UTR. (A and B) RNA was isolated at the indicated times after actinomycin D application to 293 cell lines, and the stability of NOLC1 in three independent experiments was normalized to the values for GAPDH mRNA. See also Fig. S4. Error bars indicate the SD; *P < 0.05; **P < 0.01; ns, not significant. (C and D) mRNA binding of NOLC1 for CSIG or IgG determined by RNA‐immunoprecipitation assay and analyzed by qRT–PCR and RT–PCR. GAPDH served as a negative control. We separated 1% of the total cell lysate as input, and the remainder was incubated with the antibodies. For the qPCR analysis, the input was established per unit and IgG and CSIG were used as the relative input. (E–H) RNA pulldown assays were conducted to evaluate the interaction between CSIG and NOLC1 mRNA, whole 293 cell lysate was used. Schematic diagrams of NOLC1 mRNA are shown in E and G. (I) Flag‐CSIG was purified for RNA pulldown. (J) RNA pulldown with purified Flag‐CSIG and 5′UTR, coding region (CR) and 3′UTR of NOLC1 mRNA. (K and L) 5′UTR and 3′UTR of NOLC1 mRNA were cloned into the pGL3‐promoter vector and transfected into 293 cells. The effect of CSIG knockdown on luciferase activity was measured via luciferase or Western blotting assays. (M and N) Different fragments of NOLC1 mRNA 5′UTR were cloned into the pGL3‐promoter vector and transfected into 293 cells. The effect of CSIG knockdown on luciferase activity was measured with Western blotting or luciferase assays. Error bars indicate the SD; *P < 0.05; **P < 0.01; ns, not significant.

Considering the correlation between CSIG and NOLC1 mRNA, we wonder whether CSIG have any impact in NOLC1 transcription. To verify our hypothesis, we conducted a luciferase analysis with the NOLC1 promoter (Gao *et al*., 2011) cloned into the pGL3‐basic vector, while clear differences from the mock vector were not observed (Fig. S2A and B), and chromatin immunoprecipitation (CHIP) in this region was negative (Fig. S2C and D). We then evaluated the precursor and mature mRNA of NOLC1, and the results showed that the mature NOLC1 mRNA obviously increased and the precursor mRNA did not change after CSIG knockdown (Fig. S2E). An analysis of the nuclear and plasma NOLC1 mRNA levels showed that CSIG knockdown increased both nuclear and cytoplasm NOLC1 mRNA levels (Fig. S2F), whereas the knockdown of CSIG significantly extended the half‐lives of NOLC1 mRNA compared with those observed in the control (Fig. 2A). Additionally, the half‐life of NOLC1 mRNA was shorter after CSIG overexpression (Fig. 2B), which indicated that CSIG knockdown‐induced NOLC1 mRNA turnover was dependent on NOLC1 mRNA destabilization.

### CSIG interacts with the 5′ UTR of NOLC1 mRNA

A previous report indicated that CSIG is a nonclassical RNA binding protein (Castello *et al*., 2012); thus, we wondered whether CSIG binds to NOLC1 mRNA and whether the destabilizing effect of CSIG on NOLC1 mRNA was dependent on its mRNA binding ability. Definitely, our immunoprecipitation (R‐IP) results showed that the NOLC1 mRNA immunoprecipitated with CSIG was enriched compared with IgG (Fig. 2C and D). In the RNA pulldown assays, biotinylated fragments of the NOLC1 mRNA 5′UTR and the coding region (CR) as well as the 3′UTR fragments C, D, and E (Fig. 2E) were used as probes to test the ability of CSIG to interact with NOLC1 mRNA. Whole 293 cells lysate or purified flag‐CSG (Fig. 2I) were prepared and used for the pulldown analysis as previously described (Chang *et al*., 2010). As shown in Fig. 2F and J, the 5′UTR fragment interacted with CSIG, whereas the 3′UTR and CR fragments did not, which indicated that CSIG was capable of associating with the NOLC1 5′UTR. To further identify the critical fragment for the interaction with CSIG, a series of small fragments of the NOLC1 5′UTR (Fig. 2G) were transcribed and RNA pulldown assays were performed using these biotinylated transcripts and 293 cell extracts. The interaction of the full‐length NOLC1 5′UTR with CSIG served as a positive control. As shown in Fig. 2H, CSIG proteins were detected in the pulldown materials of fragment A but not those of fragments A1–A4. Furthermore, the fragment of the 40‐base pair upstream of the ATG also showed a positive signal in RNA pulldown assay, which indicated that CSIG interacted with the NOLC1 5′UTR and was dependent on the 40‐base pair upstream of the ATG on the NOLC1 mRNA.

### Interaction between CSIG and NOLC1 5′UTR determines the ability of CSIG to destabilize NOLC1 mRNA

Next, we wondered whether CSIG knockdown affected the turnover of NOLC1 mRNA by the association of CSIG with the 5′UTR of NOLC1. A series of pGL3 basic luciferase reporter constructs bearing the NOLC1 5′UTR CR, C, D, and E of the 3′UTR fragments (Fig. 2E) and the shorter fragments of the 5′UTR A1–A4 (Fig. 2G) were designed. As shown in Fig. 2K–N, the RNAi‐mediated knockdown of CSIG was correlated with the increase in the expression of the luciferase‐encoded reporter enzyme bearing the full‐length 5′UTR of the NOLC1 mRNA. The results indicated that CSIG was capable of interacting with the 5′UTR of NOLC1 and destabilizing the NOLC1 mRNA, and this interaction was dependent on the 5′UTR, especially the 40‐base pair fragment upstream of the NOLC1 translation initiation site.

### Up‐regulated NOLC1 induced by CSIG forms a ring structure like NOLC1 overexpression

**Figure 3:**
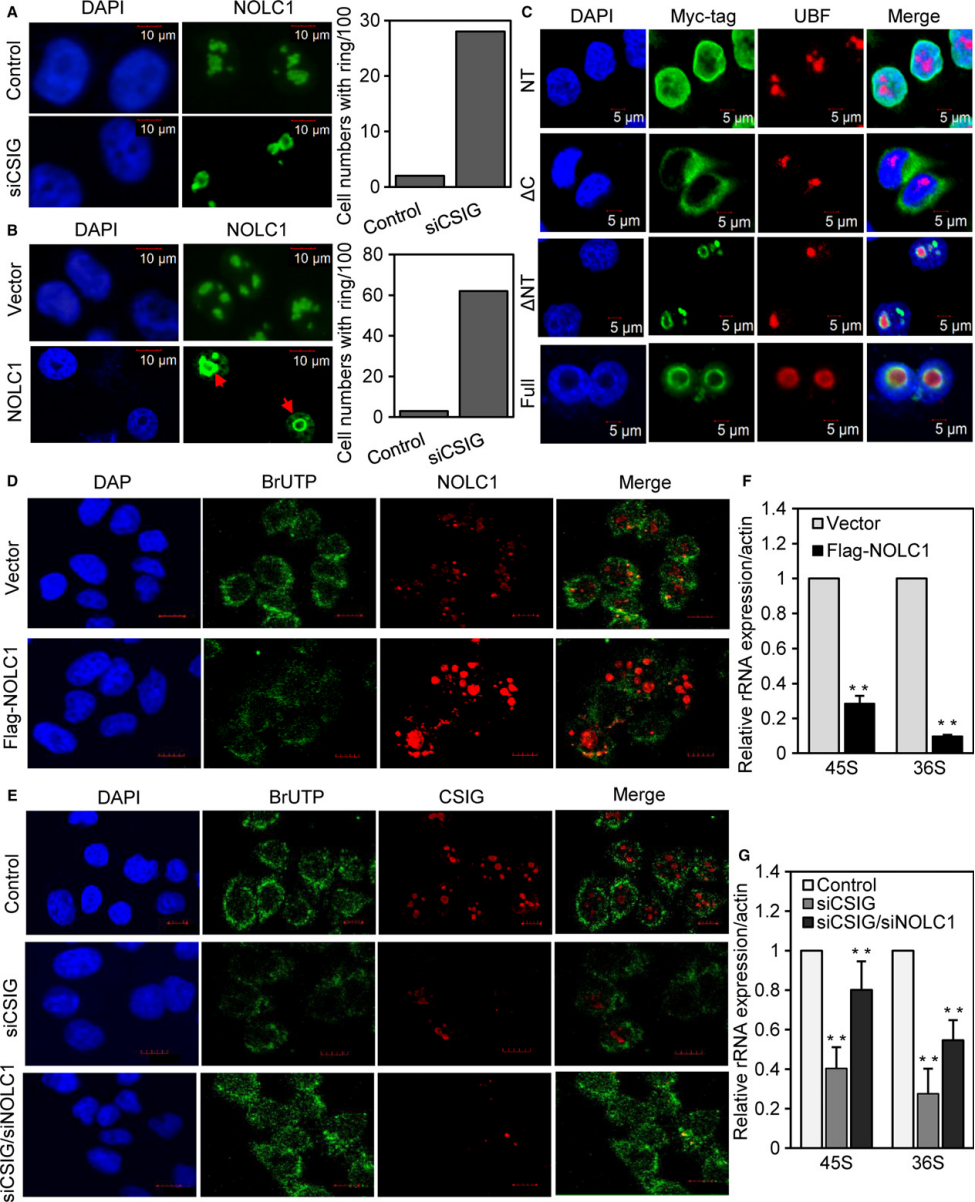
NOLC1 C‐terminus plays a critical role in the formation of rings and enhanced NOLC1 inhibited rRNA synthesis (A) CSIG knockdown‐induced rings (red arrow), the cell numbers with rings were counted in 100 total cells (right). (B) The ectopic expression of NOLC1 induced rings, the cell numbers with rings were counted in 100 total cells (right). (C) IF staining was performed after the NOLC1 truncations were transfected for 48 h with Myc‐tag (UBF was stained as the nucleolar marker). (D and E) Newly synthesized rRNA was measured by the incorporation of BrUTP after incorporated for 20 min. IF staining was performed using the indicated antibodies. Scale bar, 10 μm (also applied for Fig. S4B and C). (F and G) Pre‐rRNA level was analyzed by qRT–PCR after transfection of siRNA or Flag‐NOLC1 in the 293 cells. Random primers were used for the first‐strand cDNA. Error bars indicate the SD; *P < 0.05; **P < 0.01.

Overexpression of NOLC1 has been reported to result in the formation of spherelike structures (Chen *et al*., 1999). Because of the critical role of the nucleolus in rRNA synthesis, we wondered whether the endogenous increased levels of NOLC1 could form structures that are similar to the structures formed by exogenous NOLC1. An indirect immunofluorescence (IF) method was applied after transfection of CSIG siRNA for 72 h, and the results showed that NOLC1 labeling was enhanced after CSIG knockdown compared with that in the control, and spherelike structures were observed in nucleolus regions in the presence of increased NOLC1 expression (Fig. 3A), which is the same with what we found for NOLC1 overexpression (Fig. 3B).

### NOLC1 C‐terminus plays a critical role in the formation of rings and in rRNA processing

To identify the domain that plays a role in the formation of this special ring structure, we generated constructs of different fragments of NOLC1 using the pCDNA3.1 plasmid as described in Fig. S3. IF staining was performed following the overexpression of these constructs, and the distribution of the fragments showed that only the C‐terminus of NOLC1 could form ring structures (Fig. 3C), which indicated that this domain is indispensable for the formation of rings.

### CSIG knockdown suppresses rRNA synthesis, and it can be rescued by NOLC1 siRNA

Because of the critical role of nucleolus in rRNA transcription and related processes, we wondered whether the enhanced NOLC1 affected rRNA synthesis. A nonisotopic high‐resolution investigation of the kinetics of rRNA in living cells was performed as described before (Marc *et al*., 2008). Interestingly, NOLC1 overexpression could significantly decrease rRNA synthesis (Fig. 3D). Additionally, the newly synthesized rRNA was decreased after CSIG knockdown (Fig. 3E and Fig. S4C), which is consistent with the expression of exogenous NOLC1 as well as with the treatment of actinomycin D (Fig. S4B); also, the inhibition of rRNA synthesis induced by CSIG knockdown could be rescued by down‐regulation of NOLC1 (Fig. 3E last lane). Besides, we analyzed the pre‐rRNA level by qRT–PCR (Fig. 3F and G), which also indicated that enhanced NOLC1 might disturb rRNA synthesis.

### NOLC1 interacts with multiple proteins in the nucleolus, and enhanced NOLC1 causes nucleolar retention of NOG1

**Figure 4:**
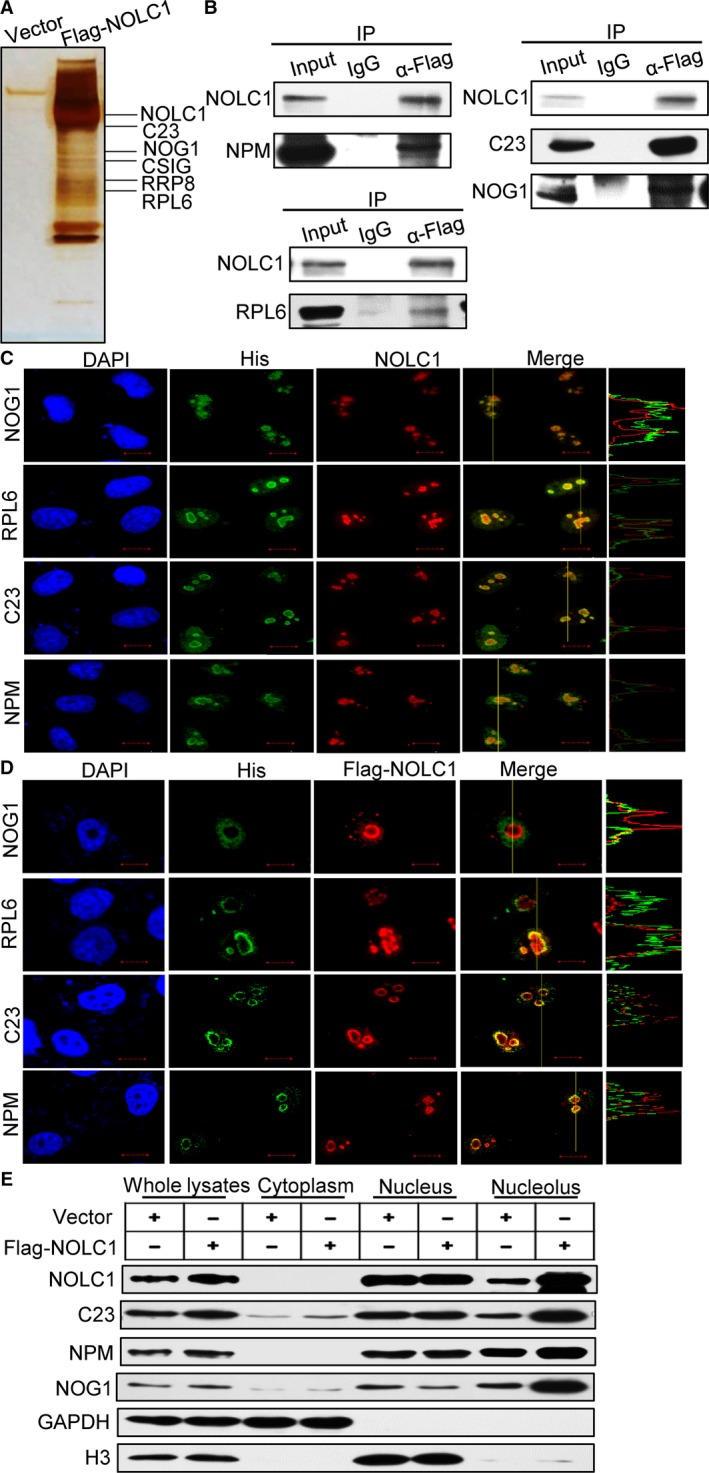
Ectopic expression of NOLC1 alters the localization of nucleolar proteins, especially NOG1. (A) Silver staining and mass spectrometry analyses of NOLC1 interacting proteins. (B) 293 cell lysates were used for co‐immunoprecipitation to confirm the interaction between NOLC1 and the proteins NOG1, NPM, RPL6, and nucleolin. (C and D) Localization of NOG1, NPM, RPL6, and nucleolin were detected in the control and NOLC1‐overexpressing cells. Scale bar, 10 μm. (E) Different subcellular compartments were isolated after NOLC1 overexpression, and the partitioning of NOLC1, NOG1, NPM, and nucleolin was assessed by a Western blot assay using the indicated antibodies.

In an effort to better understand the mechanistic roles of NOLC1 in rRNA synthesis, we employed affinity purification and mass spectrometry to identify cellular proteins that are associated with NOLC1. Various nucleolar proteins were detected in the NOLC1‐co‐eluted complexes (Fig. 4A), and some of these proteins were confirmed by IP (Fig. 4B). Considering the special ring structures formed by the enhanced expression of NOLC1, we were interested in determining whether NOLC1 overexpression could influence the distribution of these proteins. Confocal microscopy showed that nucleolin, RPL6, NPM1 and NOG1 primarily co‐localized with NOLC1 in the nucleolus and to a lesser extent in the nucleus (Fig. 4C). While after the ectopic expression of NOLC1, all these proteins interactions with NOLC1 were condensed around NOLC1 and rarely occurred in the nucleus (Fig. 4D, Fig. S5). Moreover, NOG1 expression was more specific and co‐localized with NOLC1 (Fig. 4D upper), which played a critical role in rRNA processes (Lebreton *et al*., 2008).

Next, after inducing NOLC1 overexpression, we isolated the cytoplasm, nucleus, and nucleolus. A Western blotting assay showed that the protein levels in the nucleolus increased in response to NOLC1 overexpression (Fig. 4E). In particular, the distribution of these nucleolar proteins in the nucleus decreased significantly, whereas the amount in the nucleolus increased. These data indicate that enhanced NOLC1 caused by overexpression or certain conditions, such as CSIG knockdown, could form rings capable of perturbing the distribution of certain proteins, such as NOG1, and thus disrupt rRNA synthesis. Combined with the results of the IF, we reasoned that NOLC1 caused NOG1 nucleolar detention and thus might inhibit RNA synthesis.

### CSIG decreased in older mouse liver and pancreas while NOLC1 increased

**Figure 5:**
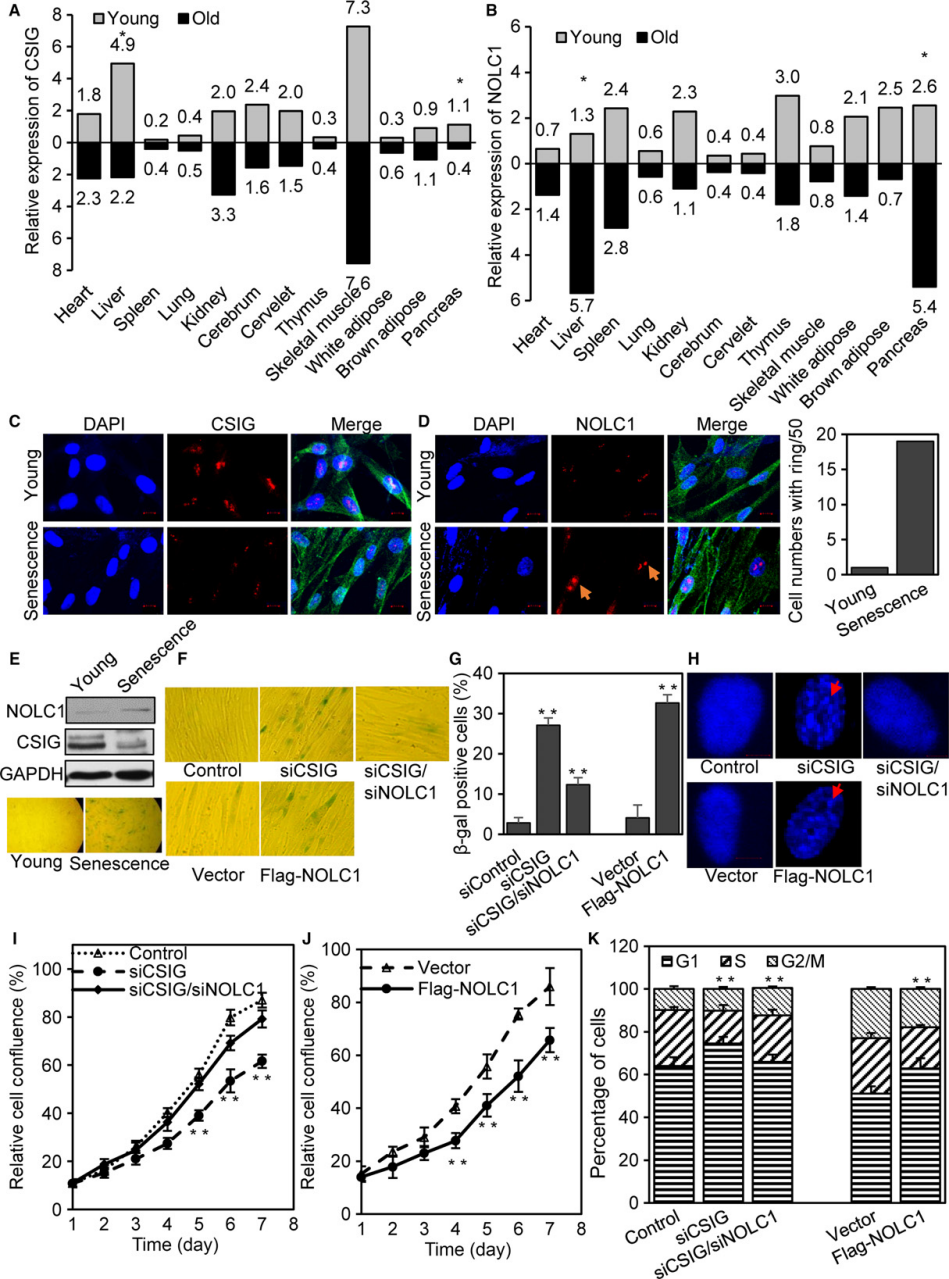
Tissue and cellular expression patterns of CSIG and NOLC1. NOLC1 is required for CSIG knockdown‐induced cellular senescence. (A and B) Total RNA of different tissues from four young litter mice and four old litter mice was extracted and used for the qRT–PCR analysis. (C) Expression of CSIG (red) in young and senescence 2BS was detected by immunocytochemistry (ICC); blue indicates the nucleus, and green indicates β‐tubulin. Scale bar, 10 μm. (D) Expression and localization of NOLC1 (red) in young and senescent 2BS were detected by immunocytochemistry (ICC); spherelike structures in senescent 2BS are indicated by a yellow arrow; blue indicates the nucleus; and green indicates β‐tubulin, the cell numbers with rings were counted in 50 total cells (right). Scale bar, 10 μm. (E) Western blot assay showing the expression of CSIG and NOLC1 in young and senescent 2BS cells. (F) 2BS cells transfected with CSIG and NOLC1 knockdown or NOLC1 overexpressing constructs were stained to assess SA‐β‐gal activity. (G) Statistical the SA‐β‐gal‐positive cells after CSIG knockdown, CSIG/NOLC1 knockdown and NOLC1 overexpression. (H) DNA DAPI staining (blue) of 2BS cells with SAHFs, which indicated that control cell or both CSIG and NOLC1 knockdown cell showed a more uniform distribution of DAPI‐DNA staining, while CSIG knockdown‐induced senescent cell showed enhanced foci (red arrow). (I and J) Cell growth assays were performed with 2BS after CSIG (or both CSIG and NOLC1) knockdown or stably transfected with Flag‐NOLC1. (K) 2BS cells were transfected with Control or CSIG/NOLC1 siRNA for 72 or 48 h after Flag‐NOLC1 transfected, and then, cell cycle analysis was conducted through flow cytometer. Data are presented as the means ± SD. *P < 0.05. **P < 0.01.

Our previous study revealed that CSIG plays an important role in cellular senescence. To further investigate the impact of the CSIG–NOLC1–rRNA pathway on aging, the expression of CSIG and NOLC1 in young and old mouse tissues was evaluated. We found that in old BABL/c mice, the expression of CSIG was decreased in liver and pancreas tissue (Fig. 5A and S6), whereas NOLC1 was up‐regulated (Fig. 5B and S6). The liver is the metabolic center of the body, and increasing amounts of data have indicated that aging and metabolic decline are closely related (Finkel, 2015), which is also consistent with the previous reports that delayed rRNA synthesis could induce cellular senescence.

### NOLC1 is increased in senescent 2BS and NOLC1 knockdown rescues CSIG‐induced senescence

Human diploid fibroblast 2BS cells have been well characterized as a model of replicative senescence (Andersen *et al*., 2005). Based on our present results in mouse tissue, we propose that the effect of CSIG on cell senescence may depend on the NOLC1–rRNA pathway. We detected the expression of CSIG and NOLC1 using Western blot and IF analyses, and our results showed that CSIG was decreased while the expression of NOLC1 was increased in senescent 2BS (Fig. 5C–E). We also observed the ring structure in senescence cells although it was not as much and distinctive as NOLC1 overexpression which we think that it is because the increase in NOLC1 in senescent cells was not as dramatic as exogenous expression of Flag‐NOLC1. Further studies showed that NOLC1 overexpression could increase the activity of SA‐β‐gal, and knockdown of NOLC1 repressed the enhanced SA‐β‐gal activity induced by CSIG knockdown (Fig. 5F and G). We also observed enhanced senescent‐associated heterochromatin foci (SAHF) after CSIG knockdown or NOLC1 overexpression (Fig. 5H).

Cellular senescence is a terminal growth arrest in the G1 phase of the cell cycle; to further investigate the role of NOLC1 on cell senescence, we detected the cell proliferation and cell cycle process in 2BS after NOLC1 knockdown and overexpression. As shown in Fig. 5I, J and K, NOLC1 knockdown could rescue the cell proliferation inhibition and cell cycle arrest induced by CSIG ablation, and the overexpression of NOLC1 repressed the cell proliferation and promoted cell cycle arrest in 2BS cells.

### NOLC1 is down‐regulated in HCC tissues and the ectopic expression of NOLC1 inhibits HCC cell proliferation

**Figure 6:**
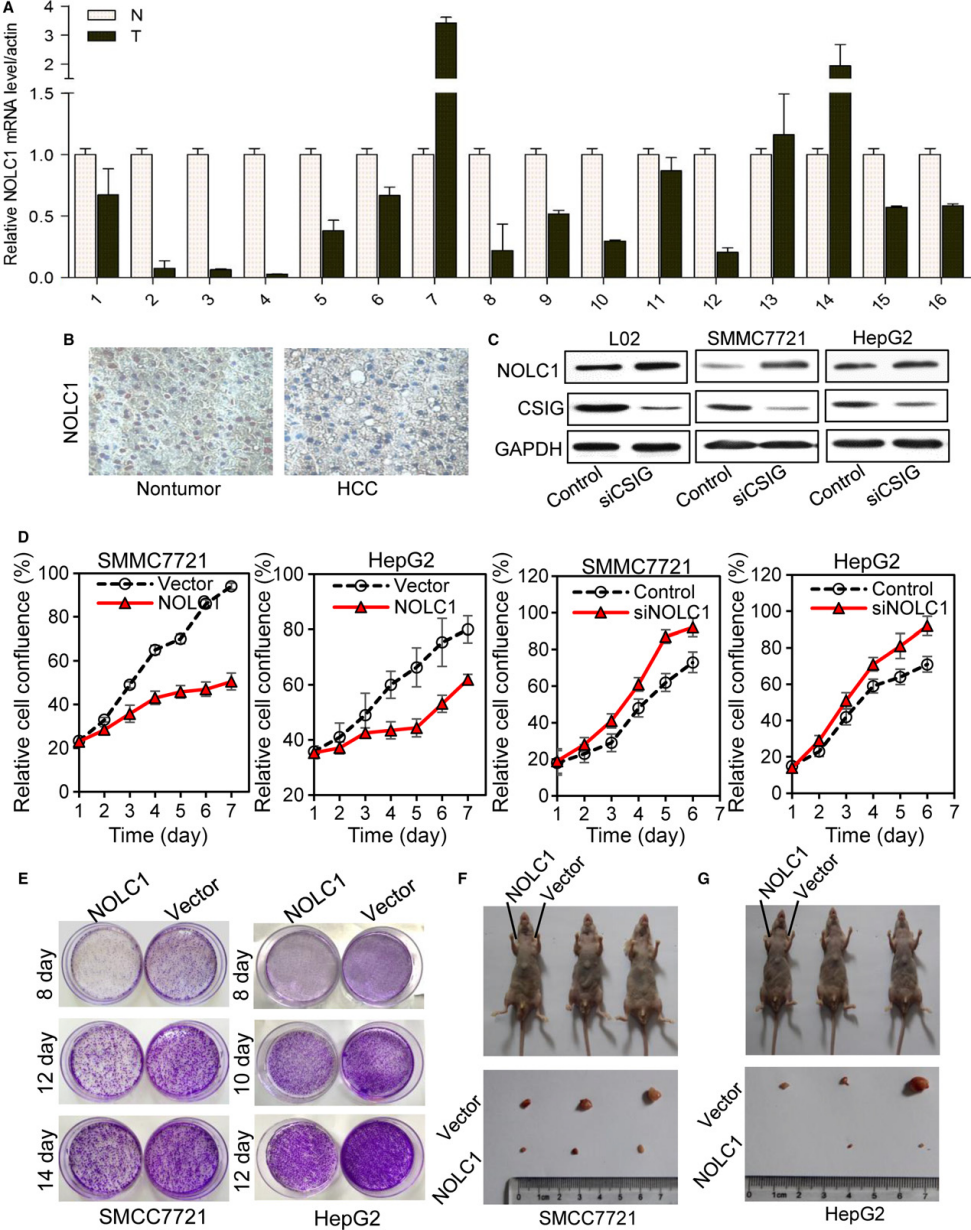
NOLC1 levels are decreased in HCC, and the ectopic expression of NOLC1 represses HCC cell proliferation and tumor growth. (A) qRT–PCR analysis of the expression of NOLC1 in 16 paired clinical specimens, N: nontumor, T: tumor. (B) Representative images of IHC confirming the expression of NOLC1 in HCC tissues and surrounding samples. (C) CSIG knockdown up‐regulates the expression of NOLC1 in SMMC7721 and HepG2 cell lines, as determined by Western blotting. (D) Cell growth assays were performed with stably transfected NOLC1 or NOLC1 knockdown. (E) Colony formation assays of SMMC7721 and HepG2 cells with NOLC1 overexpression. (F and G) Tumor growth decreased in SMMC7721 and HepG2 cells stably transfected with NOLC1.

Hepatocellular carcinoma is the most common type of primary liver cancer and the third leading cause of cancer deaths worldwide (El‐Serag, 2011). In our previous research, we found that the expression of CSIG was increased in HCC tissue and promoted HCC proliferation. To determine whether the NOLC1 pathway participated in the effect of CSIG on HCC proliferation, we assessed the expression of NOLC1 in HCC tissues from 16 patients collected in our laboratory. Our results showed that the expression of NOLC1 was decreased in most of HCC tissues (13/16) compared with the surrounding tissues (Fig. 6A and B). We then knocked down CSIG in the HCC cell lines HepG2 and SMMC7721 as well as liver immortalized L02, and the results showed that the expression of NOLC1 was increased after CSIG knockdown (Fig. 6C), which indicated that the down‐regulation of NOLC1 in HCC was correlated with the higher expression of CSIG in HCC. We then monitored the proliferation of HCC cells after the stable transfection of NOLC1. Compared with the control, NOLC1 overexpression inhibited the proliferation of HCC cells, whereas NOLC1 knockdown accelerated cell growth (Fig. 6D). Furthermore, NOLC1 up‐regulation significantly decreased the colony formation of both SMMC7721 and HepG2 cells (Fig. 6E).

### NOLC1 affects tumor growth in a HCC xenograft transplantation model

As our results suggested a functional role of NOLC1 in HCC proliferation, we next investigated whether NOLC1 contributed to HCC growth *in vivo*. The SMMC7721 and HepG2 cells stably transduced with empty vector or NOLC1‐expressing lentivirus were subcutaneously injected into BALB/c nude mice, and tumor growth was monitored. At 35 days after inoculation, the mice were killed, and the mice injected with HepG2‐NOLC1 and SMMC7721‐NOLC1 cells displayed a larger burden (Fig. 6F and G), which indicated that NOLC1 may participate in HCC tumor proliferation.

## Discussion

In higher eukaryotes, the nucleolus displays a concentric arrangement of three structural components: FC, DFC, and GC. The most well‐known function of the nucleolus is ribosome biogenesis, which includes pre‐rRNA transcription, processing, and mature rRNA assembly with ribosomal proteins. The stress on the nucleolus because of the abrogated rRNA synthesis could induce cell proliferation inhibition and cell senescence (Kazuho *et al*., 2015).

Multiple functions of NOLC1 have been reported, including cell differentiation regulation, *AGP* transcription, and rRNA transcription. Although the ectopic expression of NOLC1 was reported to induce a ring structure in the nucleolus over a decade ago (Isaac *et al*., 1998, 2001), whether endogenous NOLC1 can induce such a phenomenon and the impact of increased NOLC1 on the nucleolus and its specific mechanism remain unclear.

Here, we report that CSIG knockdown up‐regulated NOLC1 and show that CSIG and NOLC1 expression was negatively correlated in mouse tissues. Further studies revealed that CSIG promoted NOLC1 mRNA degradation by binding to the 5′UTR of NOLC1 mRNA. We also assessed the mRNA half‐life of other genes that were up‐regulated after CSIG knockdown (Fig. S7A), and found that certain genes, including caspase‐7, KPNA5 and ITGB8, had longer mRNA half‐lives after CSIG knockdown (Fig. S7B–D), whereas others did not (Fig. S7E and F). These results indicated that CSIG may be a universal RNA binding protein that acts as an RNA degradation factor, although further experiments are needed to confirm this hypothesis.

Our results also showed that NOLC1 overexpression resulted in the formation of a ringlike structure, which is consistent with the results of previous studies, and we also found that the ablation of CSIG could induce ring structures similar to those observed with the ectopic expression of NOLC1, which reminded us that the endogenously up‐regulated NOLC1 may also can form ringlike structures in the nucleolus. These findings increased our interest in the biological function of these special ring structures. Considering the critical role of the ordered nucleolus on rRNA processes, we investigated whether the rings had any impact on rRNA synthesis. As expected, both NOLC1 overexpression and knockdown of CSIG inhibited the synthesis of rRNA, and the inhibition effect of CSIG knockdown on rRNA could be rescued by NOLC1 siRNA, which indicated that the decrease in CSIG knockdown‐induced rRNA was dependent on the role of CSIG in the up‐regulation of NOLC1.

To identify the domain of NOLC1 that contributes to the rings, we constructed different truncations of NOLC1. The IF images showed that only the C‐terminus of NOLC1 permitted the formation of ring structures. A mass spectrometric analysis further revealed that multiple nucleolus proteins interacted with NOLC1, and enhanced NOLC1 disturbed the distribution of these proteins. Taken together, our results showed that enhanced NOLC1 formed rings that perturbed the distribution of nucleolar proteins, and thus abrogated its function in rRNA synthesis.

Here, we observed an interesting phenomenon in which the mild knockdown of NOLC1 increased the levels of 28S and 5.8S rRNA and knockdown of NOLC1 to a very low level reversed the rRNA levels back to normal or even lower levels (Fig. S4D). Reports have indicated that the coiled domain of NOLC1 binds to RPA140 and participates in rRNA transcription, and our results indicated that the C‐terminus of NOCL1 is critical for ring formation. Thus, the basic expression of NOCL1 is necessary for rRNA transcription, whereas the increased expression of NOCL1 disturbed the distribution of nucleolar proteins, especially such as NOG1 and thus repressed rRNA processing. This phenomenon can also explain our subsequent results in which NOLC1 overexpression was found to significantly inhibit HCC cell proliferation and NOLC1 knockdown was shown to have a weaker influence on cell growth (Fig. 6D).

We previously found that CSIG plays an important role in cellular senescence, and the ribosome also has a critical role in cell senescence (Takada & Kurisaki, 2015); thus, we were interested in determining whether 2BS cell senescence induced by CSIG was dependent on the role of CSIG in NOLC1 expression. Indeed, NOLC1 expression increased while CSIG expression decreased in senescent 2BS cells. Down‐regulation of NOLC1 rescued the CSIG ablation‐induced enhancement of SA‐β‐gal activity. To further investigate the impact of the CSIG–NOLC1–rRNA pathway on organism aging, we analyzed the expression of CSIG, NOLC1 in young and old mouse tissues, and the results revealed the decreased expression of CSIG in older mouse liver and pancreas and the increased expression of NOLC1 (Fig. 5A, S6A and B).

Hepatocellular carcinoma is the most common type of primary liver cancer and the third leading cause of cancer deaths worldwide (El‐Serag, 2011); However, the underlying mechanism of HCC is complex and largely unknown. The effects of many chemotherapeutic drugs targeting rRNA synthesis or maturation have received increasing attention in recent years (Brighenti *et al*., 2015). In our previous study, we have found that the expression of CSIG is increased in HCC and promotes HCC proliferation. Here, we found that the expression of NOLC1 was down‐regulated in most of our collected human HCC tissues. CSIG knockdown increased the expression of NOLC1 in HCC cell lines, and its overexpression repressed the proliferation of HCC cell lines and affected HCC tumor growth in nude mice. These results indicated that the CSIG–NOLC1–rRNA processing pathway may be a promising strategy for HCC.

In summary, this study elucidated the previously unknown role of CSIG as an mRNA binding protein and a potentially universal mRNA destabilizing factor. Additionally, we found that the enhanced expression of NOLC1 forms a ring structure that could cause the nucleolar retention of certain proteins and repressed rRNA synthesis. Furthermore, we demonstrated that up‐regulated NOLC1 promotes cell senescence and aging and represses cell proliferation in HCC. Our results provide some new insights into the role of the CSIG–NOLC1–rRNA pathway in aging and cancer as well as the potential mechanisms underlying aging and cancer.

## Experimental procedures

### Clinical specimens and cells

Hepatocellular carcinoma tissue and liver samples from 16 Chinese patients were obtained from the Oncology Hospital of Zhengzhou University and the Tissue Bank at Peking University of Oncology as described in our previous study (Yuan *et al*., 2015). All human samples were collected in accordance with the Declaration of Helsinki, and the use of human tissues was approved by the Institute Research Ethics Committee at the two hospitals. Informed consent was obtained from all patients. The 293T, 293, U2OS, 2BS, HepG2 cells were cultured in high‐glucose DMEM supplemented with 10% fetal bovine serum (FBS) at 37 °C in 5% CO2. The LO2 and SMMC7721 cells were cultured in RPMI 1640 medium with 10% FBS at 37 °C in 5% CO2. The cell sources are provided in the Data S1.

### Animals

BALB/c mice were maintained on a daily cycle of 12‐h light and 12‐h darkness at 24 ± 1  °C and provided free access to food and water. For the tissue extraction, four young mice (3 months old) born in the same litter and four mice from an older litter (27 months) were euthanized, and the tissues were extracted and preserved in liquid nitrogen for analysis. In the tumor xenograft assay, the HCC cells (approximately 1 × 10^6^) were resuspended in 100 μL of phosphate‐buffered saline (PBS) and subcutaneously injected into the left or right side of each BALB/c nude mouse. One side was implanted with control tumor cells, and the other was implanted with NOLC1 stably transfected HCC cells. After several weeks, the mice were euthanized and the tumors were harvested. All procedures were approved by the Institutional Animal Care and Use Committee.

### Dynamics of rRNA detection

The protocol for studying the dynamics of rRNA synthesis was previously described by Marc Thiry *et al*. (2008) with minor modifications. Briefly, cells were treated with α‐amanitin (an inhibitor of extranucleolar RNA synthesis) 2 h after the monolayer density reached 50–60%. The BrUTP complex was then transfected with FuGene 6 and then cultured for 5, 10, 20 min, respectively, after the BrUTP complex was removed. The lipofected cells were fixed and labeled for immunofluorescent signal detection by confocal microscopy as described in the Data S1.

### Mass spectrometry analysis

The 293 cells were transfected with Flag‐NOLC1 or vector plasmids and collected after 48 h and suspended in lysis buffer (50 mm Tris–HCl (pH 7.4), 150 mm NaCl, 1 mm EDTA, 1 mm DTT, 0.25 mm PMSF, 0.3% NP40, cocktail). After incubation on ice for 30 min, the cells were disrupted by sonication and incubated with anti‐FLAG antibody overnight at 4 °C. The protein complexes were then captured using protein A, resolved by SDS‐PAGE, and analyzed by LC‐MS/MS at the Integrated Center for Mass Spectrometry.

### Flag‐CSIG purification

Two hundred and thirty‐nine cells were transfected with pIRES‐Flag‐CSIG, cells were harvested after 48 h, and was resuspended in 1 mL BC500 (25 mm Tris–HCl pH7.3, 500 mm NaCl, 0.5% Triton X‐100, 20% Glycerol), ultrasonic crushed the cells (5 s on, 20 s off, ×20) and centrifuged for 15 min at 4 °C 13000 g. Twenty micro litre of ANTI‐FLAG M2 Affinity Gel was added to the supernatant and incubated overnight at 4 °C. The beads were washed once with BC500 and three times with BC100 (25 mm Tris–HCl pH7.3, 100 mm NaCl, 0.5% Triton X‐100, and 20% Glycerol). CSIG protein was eluted with 20 μL 1× flag peptide at room temperature for 2 h. 10% was used for Western blot and Coomassie blue staining for identification, and the rest was conserved at −80 °C.

### Statistical analysis

The data are presented as the mean ± SD of at least three independently performed experiments. Student's *t*‐test was used for the analyses. A probability of *P* < 0.05 was considered statistically significant. A correlational analysis was performed using Spearman's method.

## Funding

This work was supported by grants from the National Basic Research Programs of China (2012CB911203 and 2013CB530801) to TJT.

## Author contributions

FWY and TJT designed the experiments and FWY performed the experiments. YZ, LWM coordinated the study. QC prepared the HCC samples. GDL prepared materials such as antibodies. TJT supervised the project. FWY and TJT wrote the manuscript.

## Conflict of interests

The authors declare no conflict of interest in correlation with this work.

## Supporting information


**Fig. S1** Verification of the microarray data by quantitative real‐time PCR.
**Fig. S2** Analyses of NOLC1 expression.
**Fig. S3** Domain organization of the NOLC1 protein and its different truncations.
**Fig. S4** Analyses of the newly synthesized rRNA.
**Fig. S5** Changes in the distribution of nucleolar proteins after NOCL1.
**Fig. S6** Heat map of the changes in CSIG (A) and NOLC1.
**Fig. S7** Analyses of the half‐life of ZNF616, CASP7, ITGB8, CXCL6 and KPNA5 after CSIG knockdown.
**Data S1** Experimental procedures.
