# Supplementary material for: Enhanced NOLC1 promotes cell senescence and represses hepatocellular carcinoma cell proliferation by disturbing the organization of nucleolus
Source: Aging Cell. 2017 May 10;16(4):726–37. doi: 10.1111/acel.12602 (PMC5506443; doi:10.1111/acel.12602)
Supplement: Supplementary file 1 — Fig. S1 Verification of the microarray data by quantitative real‐time PCR. Fig. S2 Analyses of NOLC1 expression. Fig. S3 Domain organization of the NOLC1 protein and its different truncations. Fig. S4 Analyses of the newly synthesized rRNA. Fig. S5 Changes in the distribution of nucleolar proteins after NOCL1. Fig. S6 Heat map of the changes in CSIG (A) and NOLC1. Fig. S7 Analyses of the half‐life of ZNF616, CASP7, ITGB8, CXCL6 and KPNA5 after CSIG knockdown. Data S1 Experimental procedures. [file ACEL-16-726-s001.pdf]

Table of Contents

Supplemental Figures 1-7

• Figure S1. Additional material related to Figure 1
• Figure S2. Additional material related to Figures 2
• Figure S3. Additional material related to Figures 3
• Figure S4. Additional material related to Figure 3
• Figure S5. Additional material related to Figure 5
• Figure S6. Additional material related to Figure 6
• Figure S7. Additional material related to Figure 2

Supplemental Experimental Procedures

Supplemental References

Figure S1

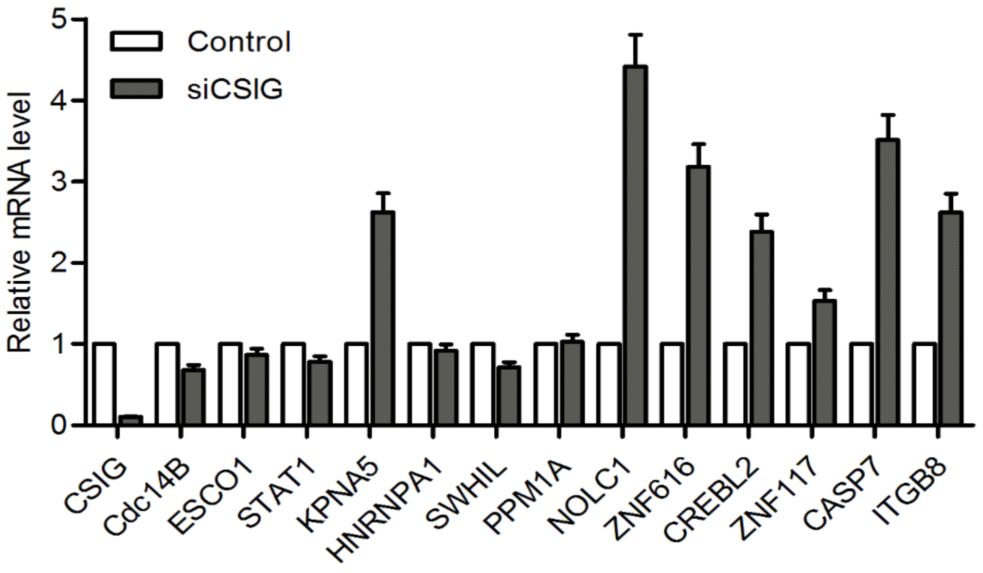

Figure S1. Additional material related to Figure 1 | Verification of the microarray data by quantitative real-time PCR. QRT-PCR analysis of gene expression after CSIG siRNA transfection for 72 h in the 293 cells. . Error bars indicate the SD.

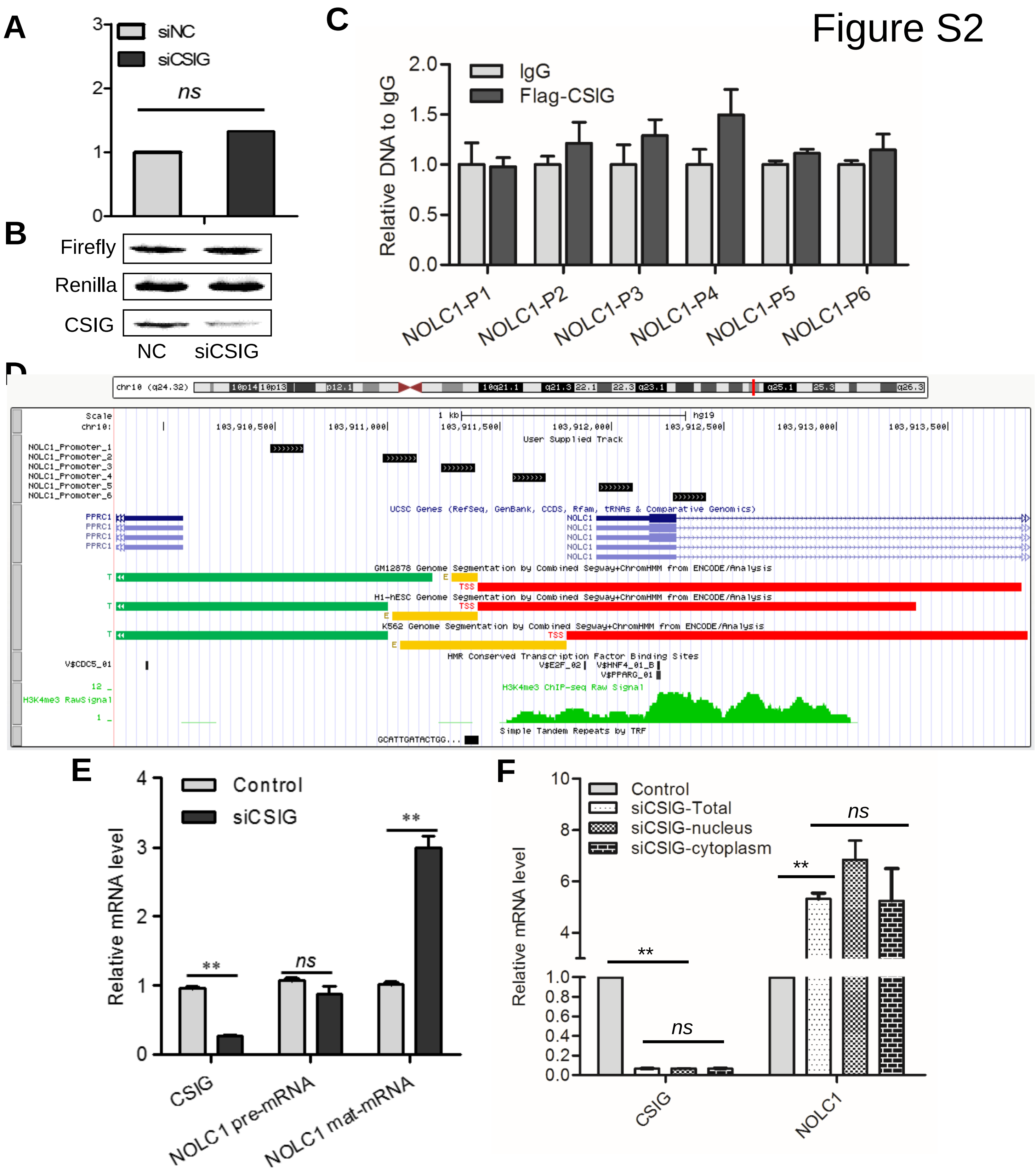

Figure S2. Additional material related to Figure 2 | A and B. NOLC1 promoter was cloned into the pGL3 basic plasmid and transfected into 293 cells after CSIG siRNA transfection for 18 h, and luciferase and western blot assays were performed to measure the activity of luciferase after plasmids transfection for 48 h. **C.** Six pairs of NOLC1 promoter primers were used for the CSIG CHIP, and the amount of precipitation was detected by q-PCR. **D.** The primer pair regions used for detecting the relative enrichment of NOLC1 promoter. **E.** Cells were harvested after transfection for 72 h, and total RNA was extracted and reverse transcribed with random primers. The pre-mRNA was measured with primers targeting the intron of NOLC1 by qRT-PCR, while primers spanning the exon junction were used for mature mRNA. **F.** Cells were harvested after CSIG siRNA transfection for 72 h, the nuclear and plasma RNA were extracted, and the expression of CSIG and NOLC1 was measured by qRT-PCR. . Error bars indicate the SD.

Figure S3

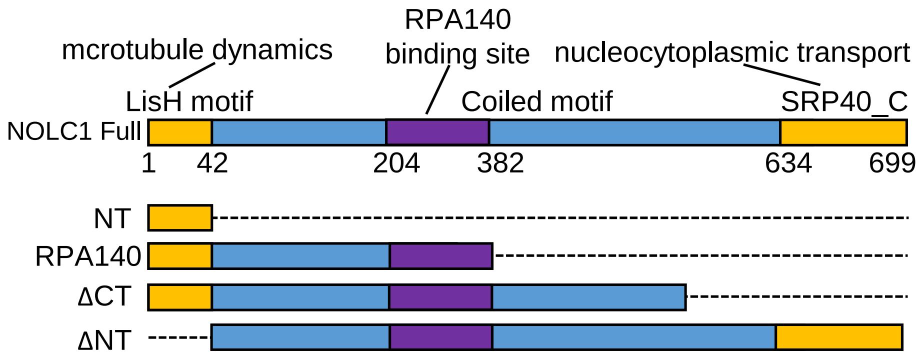

Figure S3. Additional material related to Figure 3 | Domain organization of the NOLC1 protein and its different truncations.

Figure S4

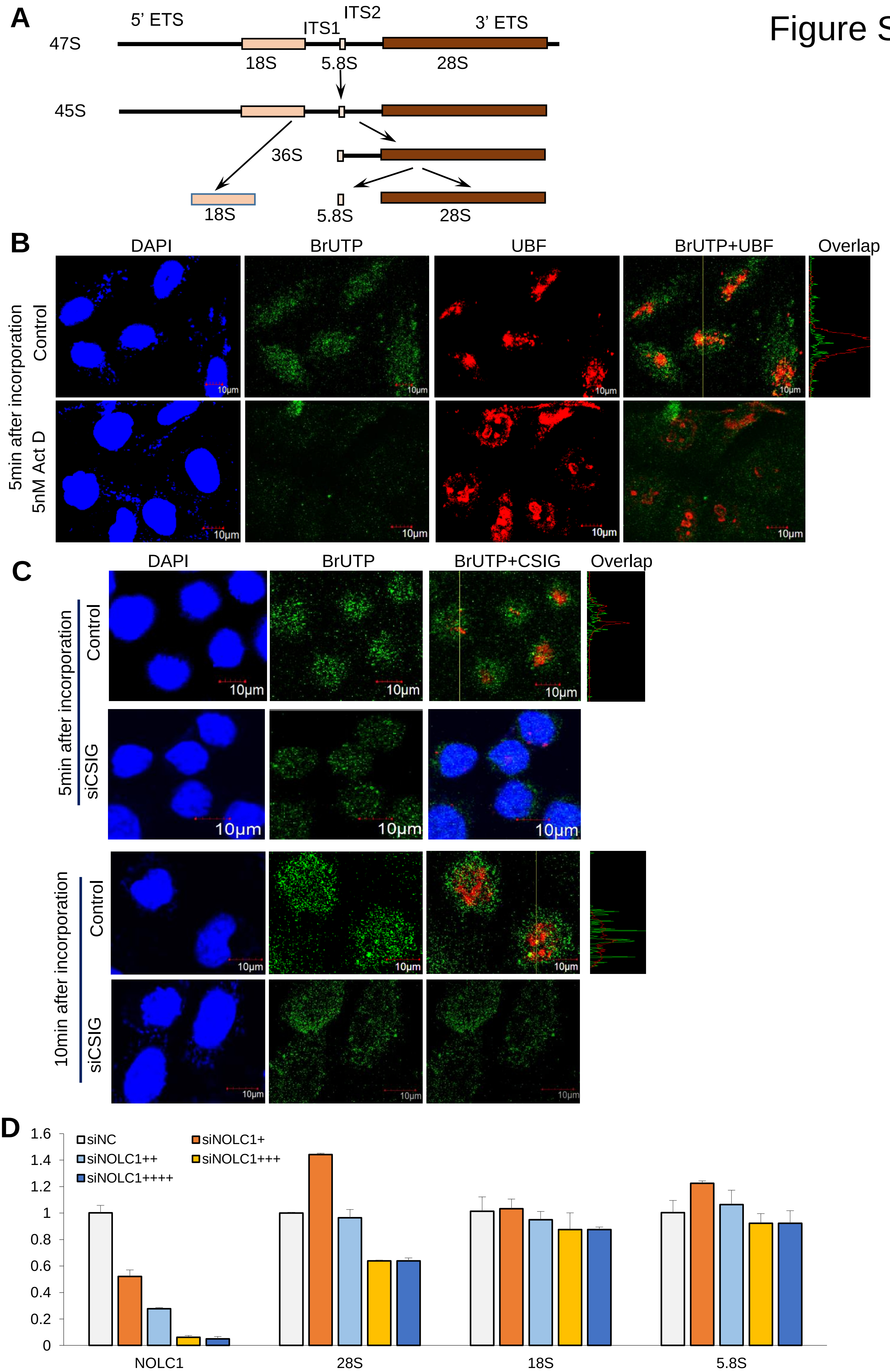
A
5' ETS
ITS1
ITS2
3' ETS
47S
18S
5.8S
28S
45S
36S
18S
5.8S
28S
B
DAPI
BrUTP
UBF
BrUTP+UBF
Overlap
5min after incorporation
Control
5nM Act D
10μm
C
DAPI
BrUTP
BrUTP+CSIG
Overlap
5min after incorporation
Control
siCSIG
10min after incorporation
Control
siCSIG
10μm
D
siNC
siNOLC1+
siNOLC1++
siNOLC1+++
siNOLC1++++
1.6
1.4
1.2
1
0.8
0.6
0.4
0.2
0
NOLC1
28S
18S
5.8S

Figure S4. Additional material related to Figure 3 | A. Schematic of rRNA processing. **B** and **C**. Newly synthesized rRNA was measured by the incorporation of BrUTP after 5nM actinomycin D treated for 6h or CSIG siRNA transfected for 72h. IF staining was performed using the indicated antibodies. Scale bar, 10 μm. **D.** QRT-PCR analysis was performed after transfection of NOLC1 siRNA (0 μM, 5 μM, 25 μM, 50 μM) for 72 h in the 293 cells, and total RNA was extracted and reverse-transcribed with random primers. . Error bars indicate the SD.

Figure S5

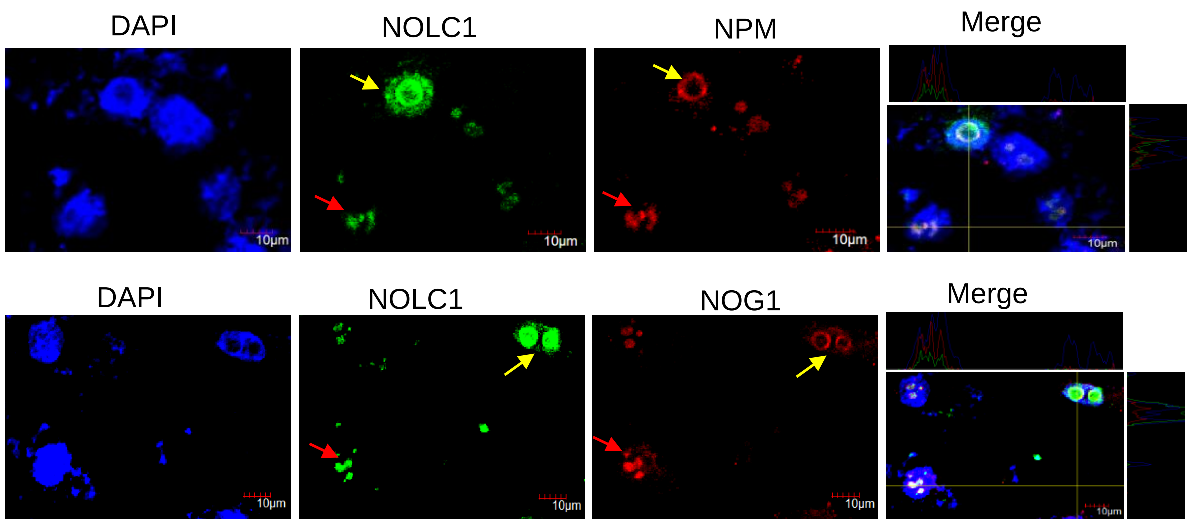

Figure S5. Additional material related to Figure 5 | Changes in the distribution of nucleolar proteins after NOCL1 overexpression (yellow arrows) compared with the control (red arrows).

Figure S6

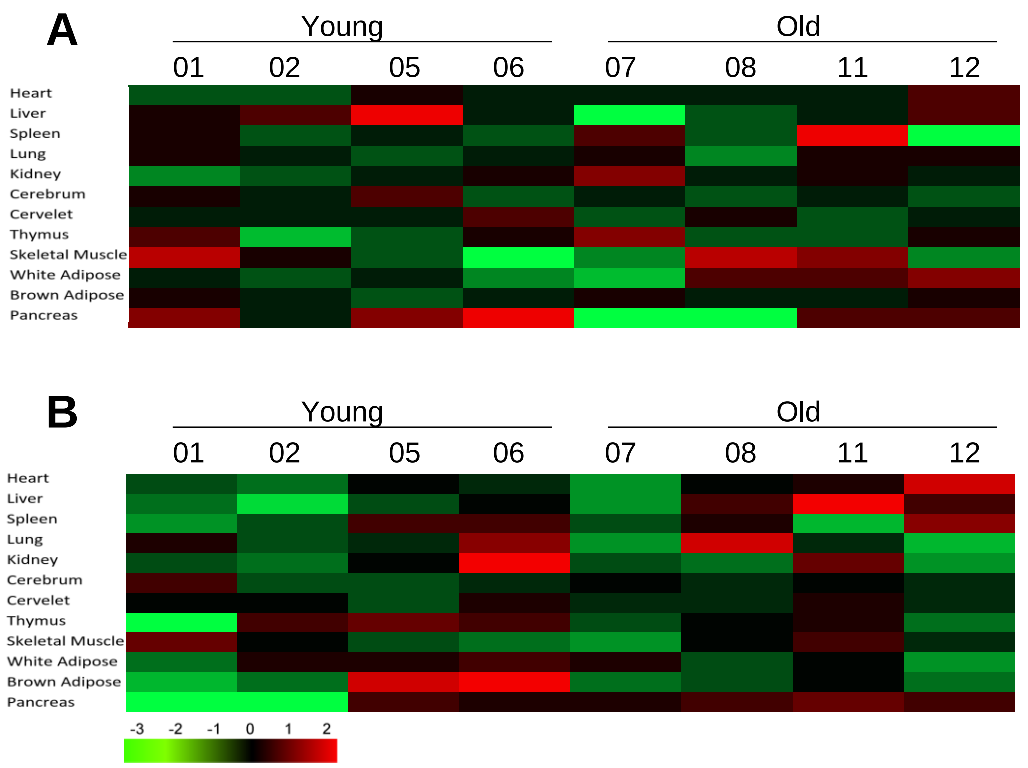

Figure S6. Additional material related to Figure 6 | Heat map of the changes in CSIG (**A**) and NOLC1 (**B**) expression in the young and old mouse tissues. HemI 1.0.3.3 software was used for this analysis.

Figure S7

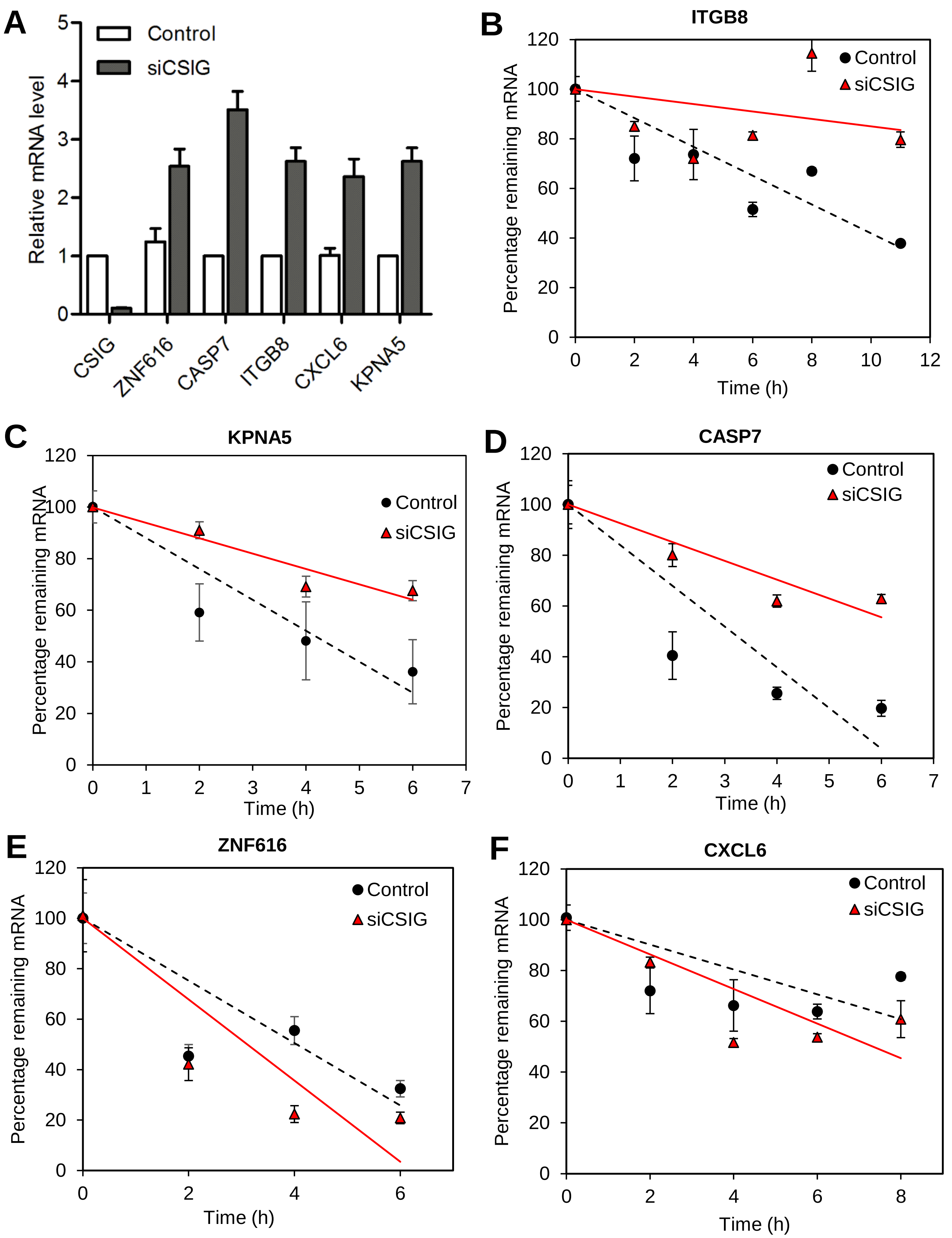

Figure S7. Additional material related to Figure 2 | **A.** Expression of ZNF616, CASP7, ITGB8, CXCL6 and KPNA5 was up-regulated after CSIG knockdown. **B-F.** Half-lives of ITGB8, KPNA5 and CASP7 mRNA were longer than the half-life of the control after CSIG knockdown, and significant changes were detected in ZNF616 and CXCL6. . Error bars indicate the SD.

Supplemental Experimental Procedures

Cell sources and transfection | Human embryonic lung diploid fibroblast 2BS cells were obtained from the National Institute of Biological Products, Beijing, China. Human liver cancer cell lines (HepG2) were obtained from the American Type Culture Collection (ATCC, Manassas, VA), and SMMC7721 was a gift from the Department of Experimental Hematology, Beijing Institute of Radiation Medicine (Beijing, China). MHCC97H were obtained from the Liver Cancer Institute of Fudan University (Shanghai, China). The human immortalized hepatocyte cell line L02 was purchased from the China Center for Type Culture Collection (CCTCC, Wu Han, China). Plasmid transfection was performed using Lipofectamine 2000 transfection reagent (Invitrogen, Carlsbad, USA). For RNAi transfection, chemically synthesized double-stranded siRNA was used. Cells were transfected with 50 nM small interfering RNA oligonucleotides (or 5-10 nM for the rescue assay against NOLC1) for 72 h using Lipofectamine RNAiMAX (Invitrogen). The siRNA sequences were as follows.

	Forward primer
Control	5′-UUCUCCGAACGUGUCACGU-3′
CSIG#1	5′-AGAAGGAACAGACCCCAGA-3′
CSIG#2	5′-AGUGGUUCUUGCAGUGCUA-3′
NOLC1#1	5′-CACCAAGAAUUCUUCAAAU-3′
NOLC1#2	5′-GCGAAAGUUACAGGCAAAU-3′
NOG1	5′-CCAGCCAUCAUGAAGAAAU-3′

Plasmids and antibodies | The full NOLC1 CDS was a type gift from prof. Hongsheng Liu, and the truncated NOLC1 was cloned into pCDNA3.1 with a Myc tag. Different fragments of NOLC1 mRNA were constructed in pGL3-enhancer plasmids, and the promoter was inserted into the pGL3-basic vector. The full-length NOG1, NPM, nucleolin, and RRP8 were inserted into pCDNA3.1 with a Myc tag, and lenti-CRISPR-v2 was acquired from Addgene (ID no. 49535) (Sanjana et al. 2014). Pspax2, pMD2G, pHBLV-puro and CSIG plasmids were maintained in our laboratory. The following single guide DNAs (sgDNAs) targeting the CSIG intron and NOG1 were cloned to CRISPR-v2 as previously described (Sanjana et al. 2014):

CSIG-sgDNA#1: ACCTCGTGGGAAGAAAAGAA

CSIG-sgDNA#2: CTCCCTCCAAACTCTAAAGA

NOG1-sgDNA: GTGATGAGCCTTCGGATTCT

The antibodies used in this study were as follows: anti-CSIG (used as previously described), anti-Myc-tag (M047-3, MBL), anti-NOLC1 (ab184550, Abcam; sc-374033, Santa Cruz Biotechnology), Anti-GTPBP4 (ab92342, Abcam), Anti-Nucleophosmin (ab52644, Abcam), anti-GAPDH (5632–1, Epitomics), anti-Flag (F1804, Sigma), anti-NPM (3542S, Cell Signaling), anti-H3 (AM8433, ABGENT) anti-BrUTP (Cell Signaling), anti-ACTIN (SC-130300, Santa Cruz Biotechnology), anti-Luciferase (Millipore), and anti-Renilla (Promega) antibody.

Lentiviral production and transfection | To generate the lentivirus, transfer plasmids were co-transfected with the packaging plasmids pMD2G and psPAX2. For each virus, 80% confluent 293T cells were transfected in OptiMEM (Life Technologies) using 10 µg of the transfer plasmid, 10 µg pMD2.G, and 10 µg psPAX2 and then transfected with CaCl2 reagent (M&C Gene Technology Ltd., Beijing, China) according to the manufacturer's instructions. After 6 h, the medium was replaced with DMEM (Life Technologies) supplemented with 10% FBS (Hyclone). After 48 h, the viral supernatants were harvested and centrifuged at 3,000 rpm at 4°C for 10 min to pellet the cell debris. The supernatant was filtered through a 0.45-µm low protein-binding membrane (Millipore) and used immediately for cell infection in the presence of 8 µg/ml Polybrene (Invitrogen, Carlsbad, USA). Pools of stable transformants were obtained by sustained selection for 3-5 days with 1 µg/ml puromycin (Invitrogen, Carlsbad, USA).

Mass spectrometry analysis | The 293 cells were transfected with Flag-NOLC1 or vector plasmids and collected after 48 h and suspended in lysis buffer (50 mm Tris-HCl (pH 7.4), 150 mM NaCl, 1 mM EDTA, 1 mM DTT, 0.25 mM PMSF, 0.3% NP40, cocktail). After incubation on ice for 30 min, the cells were disrupted by sonication and incubated with anti-FLAG antibody overnight at 4°C. The protein complexes were then captured using protein A, resolved by SDS-PAGE, and analyzed by LC-MS/MS at the Integrated Center for Mass Spectrometry.

Immunoprecipitation and western blotting | Cells were collected in IP lysate buffer, which contained protease inhibitor cocktail. The lysates were incubated with appropriate antibodies overnight at 4°C and then protein-G Sepharose beads (Millipore) for another 2 h at 4°C. The immunoprecipitates were washed 4 times with immunoprecipitation lysate buffer and resuspended in 30 µl 2x SDS loading buffer. The samples were boiled for 10 min for the western blot analysis. In the western blot assay,

proteins were extracted and separated by sodium dodecyl sulfate-polyacrylamide gel electrophoresis (SDS-PAGE). After transfer to a nitrocellulose filter membrane, the membrane was blocked with 5% nonfat milk for 1 h at room temperature and incubated at 4°C overnight with primary antibodies. After washing, the membrane was incubated at room temperature for 1 h with secondary antibody (1:5000), and the bands were visualized using ECL.

Quantitative RT-PCR (qRT-PCR) | Cellular or tissue RNA was extracted with the Rapure Total RNA Kit (Magen) following the manufacturer's instructions and reverse-transcribed with poly (dT) oligonucleotides or random primers (for rRNA or pre-mRNA analysis). SYBR Green Universal PCR Master Mix and a 7,500-sequence detection system were used (Applied Biosystems, CA, USA). The primer and probe concentrations were 0.9 μM and 0.25 μM, respectively. The PCR parameters were as follows: 95°C for 10 min and 40 cycles of 95°C for 15 s and 60°C for 1 min. The primers for PCR were as follows.

	Forward primer	Reverse primer
CSIG	GGCGGCTCTTACCCTCACTCATTG	GCCTGCTGCCTCTTCTTCTTCCTC
GAPDH	CGAGTCAACGGATTTGGTGGTAT	AGCCTTCTCCATGGTGAAGAC
KPNA5	GAGGCAAAAACCCTCCTCCA	CCTGAGGTACAATGCTGGGG
NOLC1	TTCCTGCGCGATAACCAACTC	CCTGTAACTTTCGCTCTGGGA
Pre-NOLC1	CTCGGTCGCTTACCTTCCTC	AGAACCAACGTCCTCAGTGG
45S rRNA (Carla et al., 2005)	CCTGCTGTTCTCTCGCGCGTCCGAG	AACGCCTGACACGCACGGCACGGAG
36S rRNA	GCGGAGGTTTAAAGACCC	CCAGACGAGACAGCAAAC
28S rRNA (John et al., 2015)	GCGGGTGGTAAACTCCATCT	CACGCCCTCTTGAACTCTCT
18S rRNA (John et al., 2015)	GACTCAACACGGGAAACCTC	AGACAAATCGCTCCACCAAC
5.8S rRNA (John et al., 2015)	CGACTCTTAGCGGTGGATCA	GATCAATGTGTCCTGCAATTC
5S rRNA (John et al., 2015)	TCGTCTGATCTCGGAAGCTAA	AAGCCTACAGCACCCGGTAT
NOG1	GGCTCATCGAGTGGAAACCA	AACACGTGTCTATCCGCCTC
CREBL2	TAAACGTGGTCGGAAGCCAG	TCTTCCCAGCCTTGGTATGC
mCSIG	GAAAACGCTGTTTCCCTCCG	GGAGCACTGCTAGCTAACCC
CASP7	AAGCTGACTTCCTCTTCGCC	TCCAGGTCTTTTCCGTGCTC
mNOLC1	ACCAAAGCCCCAAAGGTGAA	TTAGCTGCCTTCTGCTGGAC
ZNF616	TAAAGAAGTGCCAGTGCCCC	AGTTCAGCCAGACGTGACTC

ITGB8	GTCTGCCTGCAAAACGACCG	CCTTGGCCCAGTCCAAGAAC
ZNF117	AGTGTGGTTGAGTGTAAACAGC	AGGTGTGAAAGCATGCAAAAT
CXCL6	ACGCTGAGAGTAAACCCCAA	CCAGACAAACTTGCTTCCCG
ACTIN	GAGAAAATCTGGCACCACACC	GGATAGCACAGCCTGGATAGCAA

mRNA stability assay | Experiments were performed during the logarithmic phase of the cultured 293 or LO2 cells. The cells were treated with actinomycin D (10 μg/ml) for the indicated times, and the cultures were then washed with PBS. RNA was extracted using the RNA Extraction Kit, and first-strand cDNA was synthesized using the Transgen First Strand cDNA Synthesis kit (Transgen Biotec Co. Ltd., Beijing, China). The relative RNA amounts were analyzed by quantitative PCR.

RNA-IP and RNA pull-down | To cross-link the RNP IP complexes, the cells were exposed to UVC (400 mJ/cm2), and whole-cell lysates were prepared for immunoprecipitation using anti-CSIG or anti-FLAG. Briefly, the lysates were precleaned (30 min, 4°C) using 5 μg of IgG and 20 μl of Protein-A Sepharose beads that had been previously incubated in NT2 buffer (50 mM Tris, pH 7.4/150 mM NaCl/1 mM MgCl2/0.05% Nonidet P-40) supplemented with 5% BSA. The lysates (200 μg) were incubated (16 h, 4°C) with 20 μl of Protein-A Sepharose beads in the presence of 3 μg of antibody for 3 h at room temperature. The IP materials were washed twice with stringent buffer (100 mM Tris-HCI, pH 7.4, 500 mM LiCI, 0.1% Triton X-100, 1 mM dithiothreitol [DTT], 2 μg/ml leupeptin, 2 μg/ml aprotinin, 1 mM phenylmethylsulfonyl fluoride) and twice with IP buffer. The transcripts present in the RNP complexes were analyzed by real-time qPCR.

To perform the RNA pull-down assay, cDNA was used as a template for PCR amplification of the different NOLC1 mRNA fragments. All 5' primers contained the T7 promoter sequence (CCAAGCTTCTAATACGACTCACTATAGGGAGA). For the biotin pull-down assays, PCR-amplified DNA was used as a template to transcribe the biotinylated RNA with T7 RNA polymerase in the presence of biotin-UTP15. One microgram of the purified biotinylated transcripts was incubated with 100 μg of whole-cell lysates for 30 min at room temperature. The complexes were isolated using paramagnetic streptavidin-conjugated Dynabeads (Dynal, Oslo), and the pull-down material was analyzed by western blotting. The following primers were used.

NOLC1 3'UTR (500) F	CCAAGCTTCTAATACGACTCACTATAGGGAGAAAAACCTCAATCCTCAGT
NOLC1 3'UTR (500) R	TACACATTATAAACCAGCCAG
NOLC1 3'UTR (1000) F	CCAAGCTTCTAATACGACTCACTATAGGGAGAGCATGGACCTAACCTACT
NOLC1 3'UTR (1000) R	TACACATTATAAACCAGCCAG
NOLC1 3'UTR (1603) F	CCAAGCTTCTAATACGACTCACTATAGGGAGACCTGAGGCCATCTTCGGTGAAG
NOLC1 3'UTR (1603) R	TACACATTATAAACCAGCCAGT
NOLC1 F CR (2130) F	CCAAGCTTCTAATACGACTCACTATAGGGAGAATGGCGGACGCCGGCATTCGC
NOLC1 R CR (2130) R	TCTCACTCGCTGTCAAACTTAATAG
NOLC1 5'UTR F	CCAAGCTTCTAATACGACTCACTATAGGGAGAGCGGCCGGTGGGCTCCGCCC
NOLC1 5'UTR (230) R	CCTCCAGGCAATACGGCTC
NOLC1 5'UTR (190) R	CGACTCAGGAACCCAACAGG
NOLC1 5'UTR (150) R	TACGTCATTGCGCCGGCGCC
NOLC1 5'UTR (110) R	GCTCCACAGGGCAGTCCCAC
NOLC1 5'UTR (70) R	AGACGGGAAATCTATAAAGA
NOLC1 5’UTR (196-235)	CCAAGCTTCTAATACGACTCACTATAGGGAGATGCTGCGTCGACAACGGT AGTGACGCGTATTGCCTGGAGG

Flag-CSIG purification | 239 cells were transfected with pIRES-Flag-CSIG， cells were harvested after 48h, and was resuspended in 1ml BC500 (25mM Tris-HCl pH7.3, 500mM NaCl, 0.5% Triton X-100, 20% Glycerol), ultrasonic crushed the cells (5s on, 20s off, x20) and centrifuged for 15min at 4°C 1,2000rpm. 20μl ANTI-FLAG M2 Affinity Gel was added to the supernatant and incubated overnight at 4°C. The beads were washed once with BC500 and three times with BC100 (25mM Tris-HCl pH7.3, 100mM NaCl, 0.5% Triton X-100, 20% Glycerol). CSIG protein was eluted with 20ul 1x flag peptide at room temperature for 2h. 10% was used for western blot and coomassie blue staining for identification and the rest was conserved at -80°C。

Nucleolar extract | Cellular fractions were obtained as previously described (Chang et al., 1999). Briefly, pelleted cells were resuspended in ice-cold mild detergent buffer and centrifuged. The supernatant was retained as the cytoplasmic fraction. The pellets were then resuspended in 0.25 M sucrose/10 mM MgCl2, layered over a cushion of 0.35 M sucrose/0.5 mM MgCl2 and centrifuged. The resulting pellet was

resuspended in 0.35 M sucrose/0.5 mM MgCl2, sonicated to disrupt the nuclei, layered over a cushion of 0.88 M sucrose/0.5 mM MgCl2 and centrifuged to pellet the nucleoli. The supernatant was retained as the nucleoplasmic fraction. The nucleoli were washed by resuspension in 0.5 ml of 0.35 M sucrose/0.5 mM MgCl2 followed by centrifugation. The nucleolar pellet was resuspended in high-salt buffer and then sonicated and centrifuged. The supernatant was retained as the nucleolar extract, and the NaCl concentration was adjusted to 150 mM.

ChIP | Cells were washed twice in PBS and cross-linked in 1% formaldehyde for 15 min at room temperature. Glycine was added to 125 mM to quench the reaction. The cells were lysed in lysis buffer (1% SDS, 10 mM EDTA pH 8.0, 50 mM Tris pH 8.0, protease inhibitor complex (Roche)), incubated for 10 min at 4°C, sonicated for 45 s and then centrifuged for 10 min at 14000 x *g*. The supernatant was diluted with 4 (or 10) volumes of IP dilution buffer (1.2 mM EDTA pH 8.0, 1.1% Triton X-100, 16.7 mM Tris pH 8.0, 300 mM NaCl) and incubated with the corresponding antibody (2 mg anti-CSIG or anti-FLAG), the corresponding amount of normal rabbit or mouse IgG and 20 ml of Protein G Sepharose 4 Fast Flow beads at 4°C overnight. The beads were washed once with wash buffer 1 (0.1% SDS, 1% Triton X-100, 2 mM EDTA pH 8.0, 20 mM Tris pH 8.0, 300 mM NaCl), wash buffer 2 (0.1% SDS, 1%Triton X-100, 2 mM EDTA pH 8.0, 20 mM Tris pH 8.0, 500 mM NaCl), wash buffer 3 (500 mM LiCl, 1% NP-40, 1% Na-deoxycholate, 1 mM EDTA, 10 mM Tris pH 8.0) and washed twice with wash buffer 4 (1 mM EDTA, 10 mM Tris pH 8.0). Elution and cross-link reversal were performed at 65°C overnight in 1% SDS, 0.1 M NaHCO3, 0.5 mM EDTA pH 8.0, 20 mM Tris pH 8.0, 10 mg DNase-free RNase (Roche) or 0.5 M NaOH. For the DNA extraction, a QIAquick PCR Purification kit (Qiagen) was used. The following primers were used.

	Forward primer	Reverse primer
P1	GCTCCGCCCTTAACCAAGAT	GACTCAGGAACCCAACAGGG
P2	TTCCTGCGCGATAACCAACT	GCAGGCTTTCTCTGGTCACT
P3	CTCGGTCGCTTACCTTCCTC	ACCAACGTCCTCAGTGGAAC
P4	GGGCAGCTTGGGAAATCAAC	GCCCTTCATCTTCACTGCCT
P5	GGCATTAGTGGGAGGTAGCC	AACCCTTCTTCCTCCGGGAT
P6	GCACAGCAGGTGCTCAAATG	CACAGTTCCCTCTTCCCCAC

Cell proliferation assays and SA-β-gal assay | For the cell growth assay, the HCC cell lines SMMC7721, HepG2 and L02 with stably transfected NOLC1 were cultured in 12-well plates, and the relative growth was measured each day for 7 days. Colony-forming assays were performed with 15,000 cells after the stable transfection of NOLC1. The cells were cultured in DMEM containing 10% FBS at 37°C in 5% CO2 for 8–16 days. The cells were washed twice with PBS, fixed in 1% methanol for 30 min and then stained with 0.1% crystal violet. SA-β-gal staining was performed as previously described (Ma *et al* 2008).

Fluorescence | 293 or U2OS cells were seeded in 12-well plates for 24 h and then fixed in 4% paraformaldehyde solution for 10 min. The cell membrane was permeabilized using Triton X-100. The required antibodies were added and incubated overnight at 4°C. Secondary goat anti-mouse or anti-rabbit IgG antibodies conjugated to rhodamine (TRITC) or fluorescein isothiocyanate (FITC) were applied for 1 h at room temperature, and then DAPI was added. The cells were visualized using a laser confocal microscope (Leica TCSNT SP2, Germany).

Supplemental References

Sanjana NE, Shalem O, Zhang F. (2014). Improved vectors and genome-wide libraries for CRISPR screening. Nat Methods. 11, 783-4.

Carla Grandori, Natividad Gomez-Roman, Zoe A. Felton-Edkins, Celine Ngouenet, Denise A. Galloway, Robert N. Eisenman and Robert J.White. (2005). C-Myc binds to human ribosomal DNA and stimulates transcription of rRNA genes by RNA polymerase I. Nat Cell Biol. 7, 311-8.

John G. Gibbons, Alan T. Branco, Susana A. Godinho, Shoukai Yu, and Bernardo Lemos. (2015). Concerted copy number variation balances ribosomal DNA dosage in human and mouse genomes. Proc Natl Acad Sci U S A. 112, 2485–2490.

Ma L, Chang N, Guo S, Li Q, Zhang Z, Wang W *et al*. (2008). CSIG inhibits PTEN translation in replicative senescence. Mol Cell Biol. 28, 6290–6301.
